# Exposure of intestinal explants to NX, but not to DON, enriches the secretome in mitochondrial proteins

**DOI:** 10.1007/s00204-022-03318-x

**Published:** 2022-06-08

**Authors:** Laura Soler, Ingrid Miller, Chloé Terciolo, Karin Hummel, Katharina Nöbauer, Manon Neves, Isabelle P. Oswald

**Affiliations:** 1 Toxalim (Research Centre in Food Toxicology), INRAE, ENVT, INP-Purpan, UPS, Université de Toulouse, Toulouse, France; 2 Institute of Medical Biochemistry, University of Veterinary Medicine Vienna, Veterinaerplatz 1, 1210 Vienna, Austria; 3 VetCore Proteomics, University of Veterinary Medicine Vienna, Veterinaerplatz 1, 1210 Vienna, Austria

**Keywords:** Deoxynivalenol, NX, Gut, *Fusarium graminearum*, Explant, Proteome

## Abstract

NX is a type A trichothecene produced by *Fusarium graminearum* with limited information on its toxicity. NX is structurally similar to deoxynivalenol (DON), only differing by the lacking keto group at C8. Because of the structural similarity of the two toxins as well as their potential co-occurrence in food and feed, it is of interest to determine the toxicity of this new compound. In this study, we compared the protein composition of the extracellular media of pig intestinal explants (secretome) exposed to 10 µM of DON or NX for 4 h compared with controls. The combination of two complementary quantitative proteomic approaches (a gel-based and a gel-free approach) identified 18 and 23 differentially abundant proteins (DAPs) for DON and NX, respectively, compared to controls. Functional analysis suggested that, whereas DON toxicity was associated with decreased cell viability and cell destruction, NX toxicity was associated with an enrichment of mitochondrial proteins in the secretome. The presence of these proteins may be associated with the already known ability of NX to induce an intestinal inflammation. Overall, our results indicated that DON- and NX-induced changes in the extracellular proteome of intestinal explants are different. The increased leakage/secretion of mitochondrial proteins by NX may be a feature of NX toxicity.

**Supplementary Information:**

The online version contains supplementary material available at 10.1007/s00204-022-03318-x.

## Introduction

Various filamentous fungi, mainly from the genera *Aspergillus*, *Penicillium,* and *Fusarium* produce secondary metabolites in food and feed known as mycotoxins that are toxic to both humans and animals (Payros et al. 2021a).

In temperate climates, infections of wheat and corn by the pathogen *F. graminearum* and related species are a chronic problem. The resulting food and feed is contaminated with the mycotoxin deoxynivalenol (DON; Miller 2016). In Europe and Asia, exposure to DON can approach or exceed the tolerable daily intake, especially in children (JEFCA 2011; Knutsen et al. 2017; Vin et al. 2020).

**Fig. 1:**
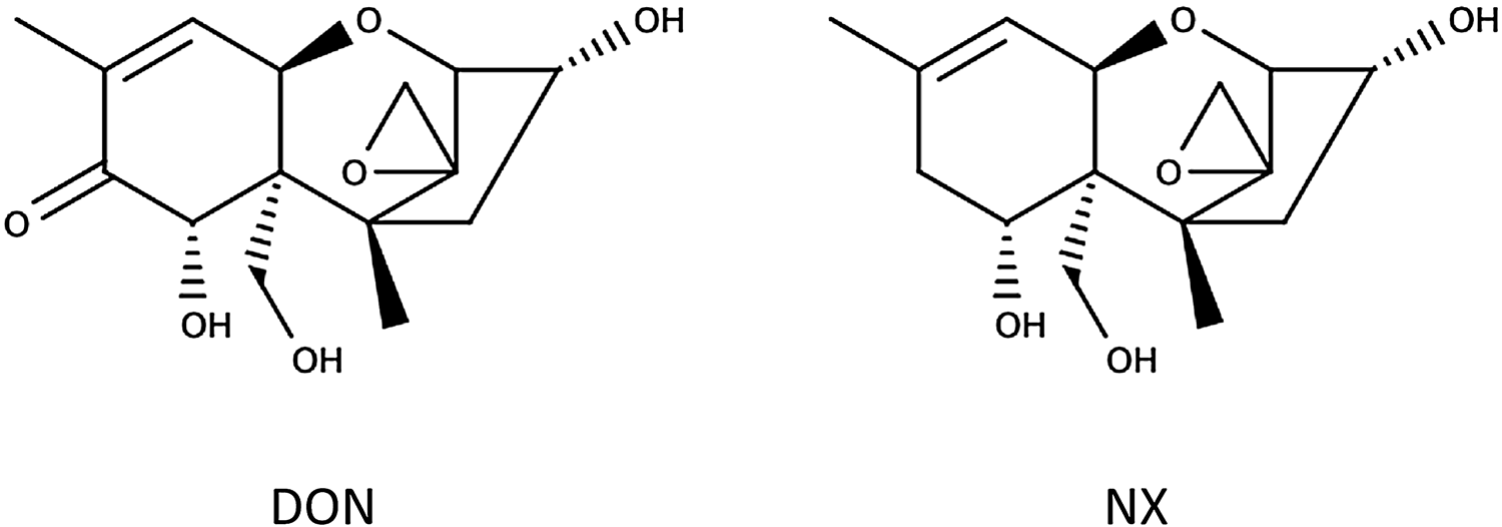
Structural formulas of the two mycotoxins in this study, DON (deoxynivalenol) and NX

A decade ago, a genetic population of *F. graminearum* was discovered that produced the novel trichothecene NX, an analogue of DON lacking the carbonyl moiety at the C8-position (Fig. 1) (Varga et al. 2015; Aitken et al. 2019; Chen et al. 2022). NX is biosynthesized by new chemotypes of *F. graminearum* populations first identified in the Midwest United States (Gale et al. 2010; Crippin et al. 2019). DON and NX have been demonstrated to co-occur. Analyses of corn samples that contained high amounts of DON have found NX at 1–7% of the DON concentration (Crippin et al. 2020).

Because it is a high human exposure toxin, much effort has been spent to understand the mechanisms of toxicity of DON. At a cellular level, DON interacts with the peptidyl transferase region of the 60S ribosomal subunit (Garreau de Loubresse et al. 2014). It induces ribotoxic stress, triggering an inflammatory cascade mediated by the activation of mitogen-activated protein kinases (MAPKs) and oxidative stress that leads to apoptosis (Pestka 2010; Lucioli et al. 2013; Payros et al. 2016). The toxic effects associated with DON exposure include alterations on protein synthesis, immune system function, and intestinal functions (Pestka 2010; Pinton and Oswald 2014). DON also interacts with several appetite suppression systems (Terciolo et al. 2018).

The toxicological characterization of NX, however, is far from complete. The few available data show that NX and DON share similar mechanisms of toxicity, including a similar inhibition of protein biosynthesis, as well as cytotoxicity, oxidative stress-related, and pro-inflammatory effects (Varga et al. 2015, 2018; Woelflingseder et al. 2018, 2020).

Recently, the comparative intestinal toxicity of DON and NX was assessed with human intestinal epithelial cells and porcine jejunal explants (Pierron et al. 2022). Both toxins had a similar impact on the viability of human intestinal epithelial cells in vitro. However, NX displayed a greater toxicity to pig intestinal explants as evidenced by the observed histopathological lesions and the results of genome-wide analysis. Gene expression data suggested that the two toxins targeted the same molecular processes namely inflammation, immune response, cell proliferation, differentiation, apoptosis, and growth. Differences between the two toxins were limited to a higher number of genes regulated per pathway as well as a higher magnitude of the gene expression changes in tissues exposed to NX (Pierron et al. 2022).

Both to assess structure–activity relationships in this class of trichothecene and the potential for DON and NX to co-occur, it is important to understand the risk associated with these toxins. Indeed, the investigation of the interaction between these molecules requires a clear understanding of the toxic impacts of each. One of the preferred approaches in clinical systems biology used to identify relevant mechanistic differences and their associated potential biomarkers is the study of tissue-specific leakage and secreted proteins (commonly referred to as the secretome) using proteomics (Hathout 2007). These are proteins that normally function inside cells, but can be released into plasma due to specific trafficking, signaling, or cell damage. The secretome includes many of the most important diagnostic markers in clinical investigations. This would include for example cardiac troponins for myocardial infarction, and liver enzymes for hepatocellular damage (Lai et al. 2010).

The objective of this study was to combine two complementary quantitative proteomic approaches (a gel-based and a gel-free method) to characterize and compare the secretomes of pig intestinal explants exposed to DON and NX with the aim of identifying specific toxic effects of each mycotoxin.

## Materials and methods

### Experimental design and statistical rationale

An ex vivo model based on pig jejunal explants was used in the present study. This model is convenient to study the molecular events that depend on interaction between the different intestinal cellular types as well as signaling gradients present along the crypt-villus axis. The model also allows for the analysis of control-treated paired explants, thus accounting individual variability. Pig intestinal explants were used as surrogates of human tissues. Pig is a good model for extrapolation to humans, as their digestive physiology is very similar to that of humans (Swindle 2012). Moreover, the intestinal explant model using post-weaning animals as tissue donors is suitable for analysis of the intestinal toxicity of trichothecenes (Kolf-Clauw et al. 2013; Alassane-Kpembi et al. 2017a, b; Graziani et al. 2019). Because the exposure time is limited to 4 h when intestinal explants are used due to the greater possibility of tissue degradation with longer incubation times, the concentrations of both toxins were set at 10 µM. This strategy is recommended in proteomic studies in toxicology (Rabilloud and Lescuyer 2015), because this concentration is high enough to give useful insights into the specific molecular mechanisms involved in toxic injury. Moreover, examination at an early time point ensures that cell mortality is low enough to avoid pollution of proteomic analysis by events that are strictly related to cell death.

Two-dimensional fluorescence difference gel electrophoresis (2D-DIGE) confirmed by immunoblotting was performed in six-paired control and treated samples. Paired t tests (GraphPad Software, San Diego, CA, USA) and paired ratios were used to analyze differences in protein abundance in all cases. Selection criteria for spots defined as differentially regulated between control and DON- or NX-treated samples from the same animal were a fold-change of  ≥ 1.5 or ≤ 0.67, presence in at least eight out of 12 gel images per group and appropriate spot quality. Differentially abundant proteins (DAPs) detected in at least one animal were listed and used for functional analysis. Label-free liquid chromatography–mass spectrometry (LC–MS/MS) was performed with three biological replicates in each group (control, NX, and DON). T tests in a nested design were performed in Proteome Discoverer 2.4 with a pairwise comparison. The significance level was set to an adjusted *p* value ≤ 0.05 and fold-change ratios of ≥ 2 or ≤ 0.5. Because fewer animals were included using this approach, only DAPs detected in all animals were listed and used for functional analysis.

### Sample preparation

DON was purchased from Sigma-Aldrich (St. Quentin Fallavier, France). NX (deacetylated product 7a-hydroxy, 3, 15-dideacetylcalonectrin), a kind gift from Dr JD Miller, was obtained as described elsewhere (Aitken et al. 2019). Toxins were dissolved in purified water and were stored aliquoted at a stock concentration of 5 mM at −20 °C. A total of six 4-week-old crossbred female piglets were used. The experiment was conducted following the guidelines of the French ministry of agriculture for animal research. All animal experimentation procedures were approved by the Ethics Committee of pharmacology–toxicology of Toulouse-Midi-Pyrénées in animal experimentation (Toxcométhique) (No. TOXCOM/0163/PP) in accordance with the European Directive on the protection of animals used for scientific purposes (Directive, 2010/63/EU). Jejunal explants were obtained as previously described (Lahjouji et al. 2020) and rinsed for 30 min in complete cell control medium. Medium free of bovine fetal serum was used to avoid polluting secretomes with foreign proteins, and included William’s Medium E (Sigma-Aldrich), glucose 4.5 g/L (Sigma-Aldrich), ITS (insulin transferrin sodium selenite) 1× (Sigma-Aldrich), alanyl-glutamine 30 mM (Sigma-Aldrich), 1% penicillin–streptomycin, and 0.5% gentamycin (Eurobio). Explants were exposed to 10 μM of DON, NX or vehicle (water) at 39 °C for 4 h under conditions described elsewhere (Alassane-Kpembi et al. 2017b; García et al. 2018). After incubation, the medium was recovered (3 mL), and debris was discarded by centrifugation at 5000*g* for 15 min. Concentrated secretomes were obtained using ultrafiltration centrifugal filter units until volumes were reduced approximately tenfold (Amicon Ultra-2 mL Centrifugal Filters 3 kDa MWCO, Merck-Millipore, Darmstadt, Germany). Total protein concentration was determined using the Bradford method (Bradford 1976) and samples were kept at –80 °C until analysis.

### 2D-DIGE analysis (gel-based approach)

Two-dimensional electrophoresis with CyDye-labeled samples (2D-DIGE) was performed as previously described (Miller 2012; Gebhard et al. 2018). Appropriate amounts of concentrated secretomes (25 µg protein per 2D gel and sample) were freeze-dried, re-dissolved in DIGE labelling buffer, and minimally labelled with CyDyes (Cytiva, Vienna, Austria). A Cy2-labelled pool of all samples was used as internal standard, Cy3 and Cy5 were used for single samples in a reverse labelling setup. The whole set contained six biological replicates per group. Per gel, a pool of two labelled samples (Cy3, Cy5) and the internal standard (Cy2) underwent isoelectric focusing in 10 cm IPG pH 4–10 NL homemade strips prior to SDS-PAGE in 140 × 140 × 1.5 mm gradient gels (*T* = 10–15%, *C* = 2.7%). Images were captured on a Typhoon RGB and evaluated with DeCyder V5.02 software (both Cytiva).

For protein identification, 2D-DIGE gels were silver stained under MS-compatible conditions (Miller 2012). Selected spots of sufficient intensity and quality (for regulation criteria see above) were excised manually. Samples were prepared and mass spectrometric analysis was performed according to Gutiérrez et al. (2019). Briefly, silver stained spots were de-stained using potassium hexacyanoferrate (III) and sodium thiosulfate. After several washing steps with ammonium bicarbonate supported by ultrasonication, proteins were reduced with dithiothreitol and alkylated with iodoacetamide. Digestion was performed with trypsin overnight. Extracted and dried peptides were dissolved in 0.1% TFA (trifluoroacetic acid) for LC–MS/MS analysis applying a 390 min LC gradient. Detailed parameters for the nano-LC Orbitrap MS/MS are provided in Gutiérrez et al. (2019).

A database search was performed using the Proteome Discoverer software 2.4.0.305 (Thermo Fisher Scientific, USA) in the UniProt database of *Sus Scrofa* (TX: 9823) as well as the cRAP database (https://www.thegpm.org/crap/). Full tryptic cleavage was allowed with a maximum of two missed cleavage sites, a precursor mass tolerance of 10 ppm and a fragment mass tolerance of 0.02 Da. For the search, carbamidomethylation (+ 57.021 Da) of cysteine as a static modification was used, whereas oxidation of methionine (+ 15.995 Da) and N-terminal acetylation (+ 42.011 Da) were set as variable modifications.

### Label-free LC–MS/MS (gel-free analysis)

For the label-free LC–MS/MS, 30 µg of each sample were digested using the FASP protocol on 10 kDa filters (Pall, New York, NY, USA) with slight modifications. The filter units were washed twice with 500 µL 8 M urea in 50 mM tris (pH 8.0) and centrifuged for 20 min at 10,000*g*, and once with 300 µL and centrifuged for 15 min at 10,000*g*. The proteins in the samples were reduced (200 mM dithiothreitol, 37 °C, 30 min) and alkylated (500 mM iodoacetamide, 37 °C, 30 min) on the filter on a thermomixer (Eppendorf, Germany). Next, the samples were centrifuged for 15 min at 10,000*g* and washed twice with 100 µL 50 mM tris (pH  8.0), and then centrifuged for 15 min at 10,000*g*. Digestion was carried out using Trypsin/LysC Mix (Thermo Fisher Scientific, Waltham, MA, USA) in a ratio of 1:25 (Protease: protein) overnight at 37 °C. Digested peptides were recovered by centrifugation for 15 min (10,000*g*) and with three times 50 µL of 50 mM tris and 15 min centrifugation each time. The resulting peptide solution was acidified with 1 µL concentrated TFA for subsequent desalting and clean-up using C18 spin columns (Pierce, Thermo Fisher Scientific) according to the manufacturer’s protocol. The dried peptides were re-dissolved in 300 µL 0.1% TFA of which 3 µL were injected to the nano-LC–MS system (Ultimate 3000 RSLC, QExactive HF, Thermo, Waltham, MA, USA).

The LC–MS/MS analysis was done as described above with just a longer gradient used. The step from 4 to 31% B was prolonged to 60 min. The MS database search was also performed as given in “2D-DIGE analysis (gel-based approach).

### Functional analysis

To assess which cell compartments/functions/pathways were mainly represented by the peptides/proteins analyzed, a system biology analysis was performed using combined lists of DAPs from the two proteomic approaches. In brief, the UniProtKB accession numbers from all DAPs were recovered and listed. From these numbers, the official human gene symbols (HuGO Gene Nomenclature Committee) were retrieved from *Sus scrofa* annotated proteins, whereas uncharacterized proteins were mapped to the corresponding *Homo sapiens* orthologs by identifying the reciprocal best BLAST hits. The list of proteins showing differential abundance was uploaded in DAVID Bioinformatic database v6.8 software (Huang et al. 2009) to obtain an overview of Gene Ontology (GO) terms for the cellular compartment, molecular function, and biological processes in which DAPs are involved. The list of proteins showing differential abundance was also uploaded onto Ingenuity Pathway Analysis (IPA) (Qiagen Bioinformatics, Hilden, Germany) and mapped to the respective databases of each tool. IPA uses networks, diseases, and molecular and cellular functions generated from previous publications and public protein interaction databases using the Ingenuity Knowledge Base as a reference.

### Immunoblotting analysis

Proteomic results were checked by immunoblotting using a cross-reacting antibody for the human protein ATP synthase F1 subunit beta (ATP5F1B; ABclonal, Woburn, MA, USA), a protein that shows differential abundance according to both 2D-DIGE and label-free LC–MS/MS analysis.

The procedure was performed as previously described (Miller et al. 2006), with modifications. Appropriate amounts of (unlabeled) samples were separated on SDS-PAGE on 10–15% T gradient gels over a separation distance of 8 cm and semi-dry blotted onto nitrocellulose (Cytiva). After fluorescence staining of the protein patterns with RuBPS (ruthenium (II) tris bathophenanthroline disufonate) and scanning on the Typhoon RGB, membranes were incubated in primary and secondary antibodies (cross-absorbed anti-rabbit IgG-HRPO, Novex, Life Technologies, Grand Island, NY, USA). Immunoreactive bands were detected by ECL (Clarity Western ECL substrate, Bio-Rad, Hercules, CA, USA) on a Vilber Lourmat FX system (Vilber-Lourmat, Eberhardzell, Germany). The overall protein staining pattern was used as a loading control and for normalization during band intensity quantification using Fiji software (ImageJ).

## Results and discussion

**Fig. 2:**
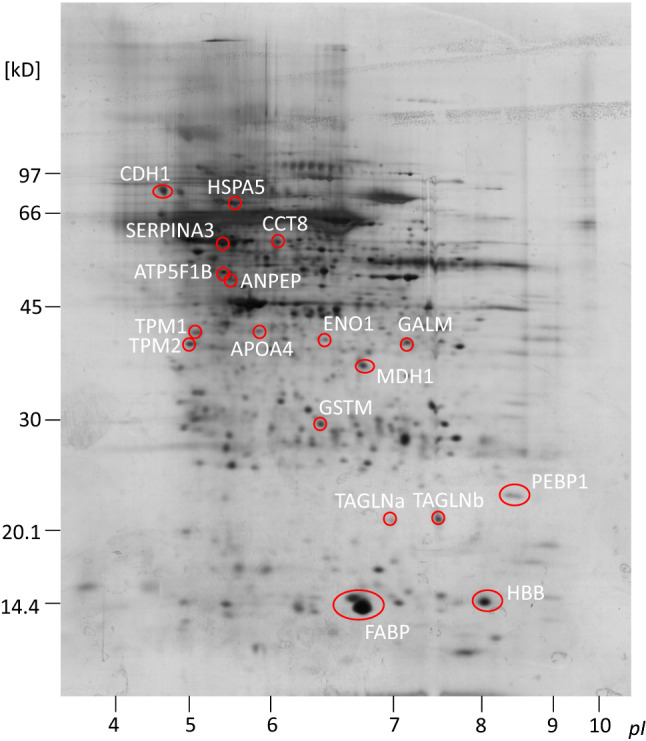
2D separation of secretome, DIGE-gel after silver staining. Shown sample: mix of secretomes (equal amounts of CyDye-labeled control, NX-treated secretome and internal standard). Red circles indicate spots found differentially abundant in comparison to control sample and were subjected to LC–MS/MS analysis for protein identification. Labels according to gene names of the respective proteins (UniProt database); for full names, see Tables 1 and 5; for regulation and identification data, see supplemental tables S1 and S2 (color figure online)

**Fig. 3:**
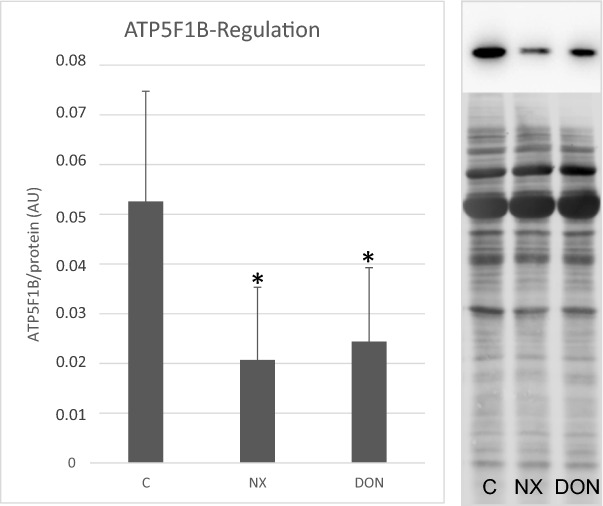
SDS-PAGE of secretomes (from tissue with/without exposure to the respective mycotoxins) and with immunoblot against anti-ATP5F1B (ATP synthase F1 subunit beta). **A** ATP5F1B band intensities in controls, NX and DON-treated supernatants (*n* = 5 per group), all normalized onto the overall protein stain of the respective lane (in AU arbitrary units); significant differences were calculated relative to untreated control (Student’s *t* test, paired, two sided; *, *p* < 0.05). **B** Blot example of one animal in immunostaining (top) and overall protein stain which was used for normalization (bottom; same lanes as shown for specific stain). **C** is for control, DON for deoxynivalenol

2D-DIGE analyses revealed differences in spot intensity between controls and DON samples in 27 matched spots, whereas 56 matched spots in NX samples differed significantly (a change of at least 50%) from controls. Of these, respectively, 15 and eight spots were positively identified by MS (Fig. 2; Supplementary Tables S1, S2). One animal was excluded from the analysis, because it showed an abnormal pattern of NX-treated samples, suggesting a technical problem. The list of differentially abundant proteins was completed with those detected by label-free LC–MS analysis, namely three proteins in DON samples and 16 in NX samples (*P* ≤ 0.05; fold-change ≥ 2 or ≤ 0.5) (Supplementary Table S3). Five proteins (APOA4, ATP5F1B, CCT8, HSPA5, and MMP1) were differentially accumulated in response to both toxins, and one of them, ATP5F1B, was found to be a DAP in both proteomic analyses (2D-DIGE and LC–MS/MS). This last protein was used to check proteomics data, and immunoblotting analysis confirmed the results (Fig. 3).

The secretomes of intestinal explants exposed to DON showed a majority of DAPs involved in structural functions (8 DAPs), together with proteins with roles in metabolism (5 DAPs) and other functions (5 DAPs, Table 1).

**Table 1:** Proteins in the secretomes of intestinal explants (*n* = 5 for 2D-DIGE; *n* = 3 for label-free LC–MS/MS analysis) which were differentially regulated due to exposure to DON (deoxynivalenol)

Technique	Official gene symbol	Name	Subcellular location		Function
Structural proteins					
2D-DIGE	CCT8	Chaperonin containing TCP1 Subunit 8	Nucleoplasm, intermediate filaments, cytosol		Involved in the transport and assembly of newly synthesized proteins, regulating transports vesicles to the cilia and ciliogenesis
2D-DIGE	TPM2	Tropomyosin 2	Actin filaments, cytosol		Actin-binding protein involved in stabilizing cytoskeleton actin filaments and receptor internalization
2D-DIGE (2 spots)	TAGLN (a, b)	Transgelin	Microtubules, mitochondria, cytosol		Actin-binding protein involved in cytoskeletal organization and cell cycle arrest
2D-DIGE	TPM1	Tropomyosin 1	Actin filaments, cytosol		Actin-binding protein involved in stabilizing cytoskeleton actin filaments and receptor internalization
2D-DIGE	CDH1	Cadherin 1	Golgi apparatus, plasma membrane, cell junctions		Calcium-dependent cell adhesion protein
2D-DIGE	APOA4	Apolipoprotein A4	Vesicles		Actin-binding protein involved in lipoproteins secretion and metabolism
LC–MS/MS	KRT14	Keratin 14	Intermediate filaments		Intermediate filaments form the cytoskeleton of epithelial cells
Proteins with a metabolic role					
2D-DIGE	MDH1	Malate dehydrogenase 1	Centrosome, Cytosol		Plays essential roles in the malate-aspartate shuttle and the tricarboxylic acid cycle, and an important role in mitochondrial NADH supply for oxidative phosphorylation
2D-DIGE	ANPEP	Aminopeptidase N	Plasma membrane		Plays a role in the final digestion of peptides generated through hydrolysis of proteins by gastric and pancreatic proteases; located in the microvillar membrane of small intestinal cells
2D-DIGE	ENO1	Enolase 1	Plasma membrane, cytosol		Glycolytic enzyme the catalyzes the conversion of 2-phosphoglycerate to phosphoenolpyruvate
2D-DIGE	GALM	Galactose mutarotase	Nucleoplasm		Enzyme that catalyzes the epimerization of hexose sugars such as glucose and galactose
2D-DIGE	ATP5F1B	ATP Synthase F1 subunit beta	Mitochondria		Mitochondrial membrane ATP synthase from Complex V
Other functions					
2D-DIGE	SERPINA3	Serpin family A member 3	Vesicles, extracellular		Serine protease inhibitor often involved in immune responses
2D-DIGE	HSPA5	Heat shock protein family A (Hsp70) member 5	Cytosol		Typical HSP70 chaperone involved in the folding and assembly of proteins in the endoplasmic reticulum and is a master regulator of its homeostasis
2D-DIGE	GSTM2	Glutathione S-transferase Mu 2	Vesicles, cytosol		Detoxification of electrophilic compounds
LC–MS/MS	MMP1	Matrix metallopeptidase 1	Vesicles, extracellular		Secreted protease involved in the degradation of interstitial collagens
LC–MS/MS	FABP4	Fatty acid-binding protein, adipocyte	Vesicles, cytosol		Lipid binding protein involved in lipid transport protein within the adipocyte

The table lists the proteomic methods used for protein detection, the official gene symbol, name, subcellular location, and function (according to Gene Ontology).

Functional analysis indicated that these proteins were either located in extracellular vesicles or were cytosolic or cytoskeletal/junctional proteins (Table 2).

**Table 2:** Functional analysis of the differentially abundant proteins (listed in Tables 1 and 5, depending on the toxin)

Gene ontology term category	DON		NX	
	Term	Benjamini	Term	Benjamini
Cellular compartment	Extracellular exosome	6.4E-5	Mitochondrial nucleoid	1.1E−2
	Cytosol	1.4E-2	Myelin sheath	7.1E−2
	Cell–cell adherens junction	5.3E−2	Mitochondrial inner membrane	7.1E−2
	Muscle thin filament tropomyosin	6.1E−2	Mitochondrial outer membrane	2.9E−1
Molecular function	Cadherin binding involved in cell–cell adhesion	1.3E−1	Antioxidant activity	2.7E−1
	Actin binding	8.1E−1	Lipid binding	7.4E−1
	Structural constituent of muscle	8.1E−1	Poly(A) RNA binding	7.4E−1
	Glycoprotein binding	9.3E−1	Transporter activity	7.4E−1
Biological process	Cell–cell adhesion	1.0E0	Hydrogen peroxide catabolic process	5.1E−1
	Toxin transport	1.0E0	Response to oxygen-containing compound	9.3E−1
	Muscle filament sliding	1.0E0	Response to reactive oxygen species	9.3E−1
	Gluconeogenesis	1.0E0	Macromolecular complex assembly	9.6E−1

Enrichment of Gene Ontology terms as a function of the cellular compartment, molecular function, and biological processes according to the DAVID bioinformatics database v6.8

The most affected molecular and biological processes were linked with cell adhesion as well as cell trafficking and shape (Tables 2 and 3). These results show that the secretomes of jejunal explants exposed to DON are markers of cell damage, loss of cell adhesion, and altered metabolism. Likewise, the top canonical pathways regulated by DON evidenced changes in glucose metabolism and enhanced detoxification (Table 4).

**Table 3:** Top canonical pathways enriched in differentially abundant proteins for each toxin according to Ingenuity Pathway Analysis (based on the protein lists in Tables 1 and 5)

Toxin	Name	*P* value	Overlap
DON	Gluconeogenesis I	6.39E−09	3.5% (6/171)
	Glutathione-mediated detoxification	2.39E−04	6.2% (2/32)
	Galactose degradation I (Leloir pathway)	3.60E−03	20.0% (1/5)
NX	Mitochondrial dysfunction	6.39E−09	3.5% (6/171)
	Sirtuin signaling pathway	1.54E−07	2.1% (6/292)
	Oxidative phosphorylation	2.56E−06	3.6% (4/11)

The overlap details the relative percentage (number) of differentially regulated proteins for each pathway, depending on the toxin

**Table 4:** Molecular and cellular functions enriched in differentially abundant proteins for each toxin according to Ingenuity Pathway Analysis (based on the protein lists in Tables 1 and 5)

Toxin	Name	*P* value range	# Molecules
DON	Cellular assembly and organization	1.36E−02 to 1.46E−06	8
	Cellular function and maintenance	1.32E−02 to 1.46E−06	13
	Cell death and survival	1.38E−02 to 5.78E−06	12
NX	Energy production	1.76E−02 to 5.74E−09	14
	Nucleic acid metabolism	1.67E−02 to 5.74E−09	12
	Small molecule biochemistry	2.28E−02 to 5.74E−09	18

# Molecules gives the number of differentially regulated proteins with the respective functions, depending on the toxin

**Table 5:** Proteins in the secretomes of intestinal explants (*n* = 5 for 2D-DIGE; *n* = 3 for label-free LC–MS/MS analysis) which were differentially regulated due to exposure to NX

Technique	Official gene symbol	Protein name	Subcellular location	Function
Mitochondrial proteins				
2D-DIGE	**ATP5F1B**	**ATP synthase subunit beta**	Mitochondria	Mitochondrial membrane ATP synthase from complex V
LC–MS/MS	HADHB	Hydroxyacyl-CoA dehydrogenase trifunctional multienzyme complex subunit beta	Mitochondria	Mitochondrial enzyme
LC–MS/MS	MAOA	Monoamine oxidase A	Mitochondria, Cytosol	Mitochondrial enzyme
LC–MS/MS	PHB	Prohibitin	Mitochondria	Mitochondrial protein that stabilizes mitochondrial respiratory enzymes and maintains mitochondrial integrity; plays a role in mitophagy
LC–MS/MS	**ATP5F1B**	**ATP synthase F1 subunit beta**	Mitochondria	Mitochondrial membrane ATP synthase from complex V
LC–MS/MS	VDAC1	Voltage-dependent anion-selective channel protein 1	Vesicles	Major component of the outer mitochondrial membrane and involved in apoptosis
LC–MS/MS	ATP5PD	ATP synthase subunit d, mitochondrial	Mitochondria	Mitochondrial membrane ATP synthase from complex V
LC–MS/MS	ALDH18A1	Delta-1-pyrroline-5-carboxylate synthase	Mitochondria	Mitochondrial enzyme
LC–MS/MS	ATP5F1A	ATP synthase F1 subunit alpha	Mitochondria	Mitochondrial membrane ATP synthase from complex V
LC–MS/MS	ATP5F1C	ATP synthase F1 subunit gamma	Mitochondria	Mitochondrial membrane ATP synthase from complex V
LC–MS/MS	SLC25A5	ADP/ATP translocase 2	Mitochondria	Mitochondrial carrier subfamily of solute carrier protein genes
LC–MS/MS	LOC100156879	Ubiquinol-cytochrome c reductase core protein 1 (90 identity)	Mitochondria	Component of the mitochondrial electron transport chain that drives oxidative phosphorylation
LC–MS/MS	PCK1	Phosphoenolpyruvate carboxykinase	Mitochondria, cytosol, endoplasmic reticulum	Cytosolic or mitochondrial enzyme involved in the regulation of gluconeogenesis
LC–MS/MS	STIP1	Stress induced phosphoprotein 1	Nucleoplasm, plasma membrane, cytosol	Regulates both the conformations and ATPase cycles of HSP70 and HSP90
Structural proteins				
2D-DIGE	**CCT8**	**Chaperonin containing TCP1 subunit 8**	Nucleoplasm, intermediate filaments, cytosol	Involved in the transport and assembly of newly synthesized proteins that regulate the transport of vesicles to the cilia and ciliogenesis
2D-DIGE	**APOA4**	**Apolipoprotein A4**	Vesicles	Actin-binding protein involved in lipoproteins secretion and metabolism
LC–MS/MS	ACTN2	Alpha-actinin-2 isoform	Actin filaments	Cytoskeletal protein found in microfilament bundles and adherens-type junctions
LC–MS/MS	DES	Desmin	Intermediate filaments	Intermediate filament connecting myofibrils to each other and to the plasma membrane
Other functions				
2D-DIGE	HBB	Hemoglobin subunit beta	Cytosol, vesicles	Oxygen transport protein also involved in the regulation of inflammation
2D-DIGE	PEBP1	Phosphatidylethanolamine binding protein 1	Plasma membrane, cytosol	Modulator of the MAP kinase, NF-kappa B, and glycogen synthase kinase-3 signaling pathways
2D-DIGE	GSTM	Glutathione S-transferase mu	Vesicles, cytosol	Detoxification of electrophilic compounds
2D-DIGE	FABP1	Fatty Acid binding protein 1	Nucleoplasm, cytosol	Lipid binding protein involved in lipid uptake and transport within the hepatocyte
2D-DIGE	**HSPA5**	**Heat shock protein family A (Hsp70) member 5**	Cytosol	Typical HSP70 chaperone involved in the folding and assembly of proteins in the endoplasmic reticulum; master regulator of its homeostasis
LC–MS/MS	**MMP1**	**Matrix metallopeptidase 1**	Vesicles, extracellular	Secreted protease involved in the degradation of interstitial collagens

The table lists the proteomic methods used for protein detection, the official gene symbol, name, subcellular location, and function (according to Gene ontology). Proteins found regulated also in DON exposure are in bold

Our results are in agreement with the known toxicity of DON that causes lesions in the intestine and the loss of the intestinal barrier (Pinton et al. 2009, 2012; Payros et al. 2021b). Indeed, the enrichment of structural proteins as well as of proteins involved in metabolism known to be present in mature enterocytes in the DON intestinal secretomes reflects increased destruction of the intestinal epithelium, and the release of cytosolic proteins into the medium. Although the pro-inflammatory role of DON is well known (Pestka 2010; Alassane-Kpembi et al. 2018; Payros et al. 2020), the intestinal secretomes contained no inflammatory proteins, probably due to the short exposure time to the toxin. Indeed, the production and release of inflammatory proteins may take more than 4 h. However, the changes observed in glucose metabolism and detoxification processes as well as changes in cellular chaperones such as HSPA5 reflect a high cellular stress, which may indeed be linked with the development of an inflammation.

The secretomes of intestinal explants exposed to NX were highly enriched in DAPs belonging to the mitochondria (14 DAPs). The list of DAPs was completed with structural proteins (4 DAPs) and proteins with other functions (6 DAPs; Table 5).

Based on the functional analysis, most of the DAPs were contained in mitochondria (Table 2) and enriched pathways were linked with mitochondrial functions (Table 5). Cellular and molecular functions connected with NX-related DAPs indicated responses to oxidative stress as well as binding of lipids and small molecules such as RNAs (Tables 2 and 3). In the present study, we observed that the changes in the secretomes of jejunal explants exposed to NX reflected alteration in cell shape and adhesion, as well as alteration in the regulation of inflammatory response. This is in accordance with previous results, showing that NX regulates genes involved in cell proliferation, differentiation, apoptosis, and growth, and particularly in immune and pro-inflammatory responses (Pierron et al. 2022). In contrast to DON, most DAPs in NX-treated samples were mitochondrial proteins. The enrichment in mitochondrial proteins of the jejunal secretomes in response to NX is likely associated with the increased inflammation caused by this toxin compared to DON. Indeed, inflammation promotes the secretion of mitochondrial content (Todkar et al. 2021). The presence of mitochondrial content in the extracellular space can mediate cell-to-cell communication and repair and function as an activator of the immune response (Miliotis et al. 2019). When contained in mitochondrial-derived vesicles, extracellular mitochondrial proteins reflect an enhanced oxidative stress and mitochondrial toxicity (Vasam et al. 2021). In our conditions, it was not possible to distinguish if the identified DAPs are free in the surrounding medium or contained in vesicles or whole mitochondria. Further analyses are needed to investigate if NX is especially toxic to cell mitochondria, and if the secretion of mitochondrial-derived vesicles is important for the increased inflammation induced by NX compared to DON.

In terms of risk analysis, the present results contribute with new information concerning the difference between DON and NX toxicity. Indeed, NX toxicity is associated with the release of extracellular mitochondrial proteins in exposed cells. Quantification of this phenomenon, in comparison with common effects such as inflammation, would be useful to characterize the combined toxicity of DON and NX and their possible interactions.

## Conclusion

Our results show that the secretomes of jejunal explants exposed to DON and NX reflect the known histological lesions as well as the metabolic changes associated with cellular stress induced by the inflammation promoted by both mycotoxins. Our results also show that NX but not DON toxicity promotes the release of mitochondrial proteins, a phenomenon possibly linked with the pro-inflammatory effects of NX. Our results further suggest that NX may be toxic to mitochondria, and call for further research on the role of mitochondria and the production of mitochondrial-derived vesicles in NX toxicity. The present results provide new evidence that DON and NX toxicity may not simply be a matter of different potencies of the same effects, contrary to what was previously thought. The comparative assessment of mitochondrial toxicity could be useful to investigate the differential toxicity of DON and NX as well as their combined effects.

## Supplementary Information

Below is the link to the electronic supplementary material.

Supplementary file1 Table S1: Proteomic evaluation of secretomes from control and mycotoxin-treated cells, investigated in 2D-DIGE approach. Ratios of paired samples (mycotoxin-treated cell supernatants/controls); bold values represent proteins with at least 50% fold-change between treatments (XLSX 14 KB)

Supplementary file2 Table S2: Identification of proteins in spots from DIGE-experiment. Spots had been selected from 2D-gels based on their regulation (see Supplemental Table S1) and proteins identified by LC-MS/MS. For position of spots in the gels see Fig. 1 (XLSX 12 KB)

Supplementary file3 Table S3: Identification and quantification of proteins in the label-free LC-MS/MS approach (one sheet per mycotoxin, starting with the significantly regulated DAPs) (XLSX 318 KB)
